# Clinical features of cryptococcosis in patients with different immune statuses: a multicenter study in Jiangsu Province–China

**DOI:** 10.1186/s12879-021-06752-x

**Published:** 2021-10-08

**Authors:** Yu Wang, Yu Gu, Kunlu Shen, Xuefan Cui, Rui Min, Siqing Sun, Chunlai Feng, Yanbin Chen, Li Wang, Min Cao, Jian Yang, Jian Yao, Jing Xu, Dang Lin, Yujian Tao, Guoer Ma, Jiaxin Shi, Bilin Chen, Yueyan Ni, Huanhuan Zhong, Yi Shi, Xin Su

**Affiliations:** 1 Department of Respiratory and Critical Care Medicine, Jinling Hospital, Medical School of Nanjing University, Nanjing, 210002 China; 2 Department of Respiratory and Critical Care Medicine, Jinling Hospital, Nanjing Medical University, Nanjing, 210002 China; 3 Department of Respiratory and Critical Care Medicine, Jinling Hospital, Southern Medical University, Guangzhou, 510000 China; 4 Department of Respiratory and Critical Care Medicine, Jiangsu Province Hospital, Nanjing Medical University, Nanjing, 210002 China; 5 Department of Respiratory and Critical Care Medicine, The Second Hospital of Nanjing, Nanjing, 210002 China; 6 Department of Respiratory and Critical Care Medicine, The Third Affiliated Hospital of Soochow University, Changzhou, 213000 China; 7 Department of Respiratory and Critical Care Medicine, The First Affiliated Hospital of Soochow University, Suzhou, 215000 China; 8 Department of Respiratory and Critical Care Medicine, Nanjing First Hospital, Nanjing Medical University, Nanjing, 210002 China; 9 Department of Respiratory and Critical Care Medicine, Nanjing Drum Tower Hospital, Medical School of Nanjing University, Nanjing, 210002 China; 10 Department of Respiratory and Critical Care Medicine, The Affiliated Jiangning Hospital of Nanjing Medical University, Nanjing, 210002 China; 11 Department of Respiratory and Critical Care Medicine, The First People’s Hospital of Nantong, Nantong, 226000 China; 12 Department of Respiratory and Critical Care Medicine, Jiangsu Province Hospital of Chinese Medicine, Nanjing, 210002 China; 13 Department of Respiratory and Critical Care Medicine, Suzhou Municipal Hospital, Suzhou, 215000 China; 14 Department of Respiratory and Critical Care Medicine, Affiliated Hospital of Yangzhou University, Yangzhou, 225000 China; 15 Department of Respiratory and Critical Care Medicine, Affiliated Hospital of Jiangsu University, Zhenjiang, 212000 China; 16 Department of Respiratory and Critical Care Medicine, The First People’s Hospital of Lianyungang, Lianyungang, 222000 China

**Keywords:** Cryptococcosis, Immunocompetent, Immunodeficient, Clinical feature, Mild-to-moderate

## Abstract

**Background:**

Current guidelines support different management of cryptococcosis between severely immunodeficient and immunocompetent populations. However, few studies have focused on cryptococcosis patients with mild-to-moderate immunodeficiency. We performed this study to determine the clinical features of pulmonary (PC) and extrapulmonary cryptococcosis (EPC) and compared them among populations with different immune statuses to support appropriate clinical management of this public health threat.

**Methods:**

All cases were reported by 14 tertiary teaching hospitals in Jiangsu Province, China from January 2013 to December 2018. The trends in incidence, demographic data, medical history, clinical symptoms, laboratory test indicators, imaging characteristics and diagnostic method of these patients were then stratified by immune status, namely immunocompetent (IC, patients with no recognized underlying disease or those with an underlying disease that does not influence immunity, such as hypertension), mild-to-moderate immunodeficiency (MID, patients with diabetes mellitus, end-stage liver or kidney disease, autoimmune diseases treated with low-dose glucocorticoid therapy, and cancer treated with chemotherapy) and severe immunodeficiency (SID, patients with acquired immunodeficiency syndrome, haematologic malignancies, solid organ transplantation or haematologic stem cell transplantation, idiopathic CD4 lymphocytosis, agranulocytosis, aggressive glucocorticoid or immunosuppressive therapy and other conditions or treatments that result in severe immunosuppression).

**Results:**

The clinical data of 255 cryptococcosis patients were collected. In total, 66.3% of patients (169) were IC, 16.9% (43) had MID, and 16.9% (43) had SID. 10.1% of the patients (17) with IC were EPC, 18.6% of the patients (8) with MID were EPC, and 74.4% of patients (32) were EPC (IC/MID vs. SID, p < 0.001). Fever was more common in the SID group than in the IC and MID groups (69.8% vs. 14.8% vs. 37.2%, p < 0.001). Of chest CT scan, most lesions were distributed under the pleura (72.7%), presenting as nodules/lumps (90.3%) or consolidations (10.7%). Pleural effusion was more common in SID group compared to IC group (33.3% vs. 2.4%, p < 0.001). Positivity rate on the serum capsular polysaccharide antigen detection (CrAg) test was higher in the SID group than in the other two groups [100.0% vs. 84.4% (MID) vs. 78.2% (IC), p = 0.013]. Positivity rate on the serum CrAg test was also higher in cryptococcal meningitis patients than in PC patients (100.0% vs. 79.5%, p = 0.015).

**Conclusions:**

The clinical presentation of MID patients is intermediate between SID and IC patients and is similar to that of IC patients. The serum CrAg test is more sensitive for the identification of SID or EPC patients.

## Introduction

The incidence of cryptococcosis in kidney transplant recipients and acquired immunodeficiency syndrome (AIDS) patients is 10.59/10,00 per year. There are one million cases of cryptococcal meningitis globally per year [1, 2]. Another study found that 32.7% of patients with cryptococcosis had no recognized underlying diseases [3]. An increasing incidence of pulmonary cryptococcosis (PC) has been identified in immunocompetent patients [4–6].

With regard to *Cryptococcus* infection, cell-mediated immunity is the predominant defense and is mainly based on *Cryptococcus*-specific Th1-type CD4+ T cells that produce interleukin-2 (IL-2), tumour necrosis factor (TNF) and interferon (IFN) [7]. A Th1–Th17 cytokine profile is associated with increased phagocytic activity and inhibition of the growth of *Cryptococcus* [7]. Our previous study revealed that patients with CD4 T lymphocyte cell counts greater than 378/µL were more likely to be asymptomatic and had a lower mortality rate than those with lower CD4+ T lymphocyte counts [8, 9]. Similarly, another study confirmed that patients with CD4+ T lymphocyte counts lower than 400/μL were prone to disseminated cryptococcosis [10]. The mortality rate in patients with low levels of CD4+ T lymphocytes was significantly higher than that in patients with normal CD4+ T lymphocyte levels [10, 11]. These reports indicated that immune status significantly influences the clinical features and outcomes of cryptococcosis.

Previous studies have mainly focused on the features of cryptococcal meningitis in severe immunocompromised patients, especially the AIDS population [12, 13]. Few studies have compared the clinical features and the performance of diagnostic methods of cryptococcosis among patients with different immune statuses in one study. The 2010 Infectious Diseases Society of America (IDSA) guidelines recommended managing cryptococcosis patients differently only based on whether they were immunocompetent or severely immunocompromised, such as AIDS and transplant patients. However, there are many patients with mild-to-moderate immunodeficiency for various underlying diseases in the clinic.

The CrAg test is playing an increasingly important role in clinical practice. According to the 2019 Revision and Update of the Consensus Definitions of Invasive Fungal Disease from the European Organization for Research and Treatment of Cancer and the Mycoses Study Group Education and Research Consortium (EORTC/MSG), a positivity test for CrAg can serve as confirmatory evidence in the diagnosis of cryptococcosis. This greatly improves the diagnostic status of serum CrAg detection.

However, previous studies mostly focused on the population with severe immunodeficiency, and few studies on the patients with immunocompetency and mild-to-moderate immunodeficiency [4, 14]. The sensitivity of CrAg in patients with different immune states remains to be further explored.

Are the clinical characteristics of mild-to-moderate immunodeficiency patients more similar to immunocompetent patients or severe immunodeficiency patients? So, based on clinical practice, we conducted this multicentre study and compared the clinical features of patients with different immune statuses.

This study compared the clinical characteristics and the value of the CrAg test in PC and EPC patients who were immunocompetent or immunodeficient (mild-to-moderate or severe immunodeficiency).

## Methods

**Fig. 1:**
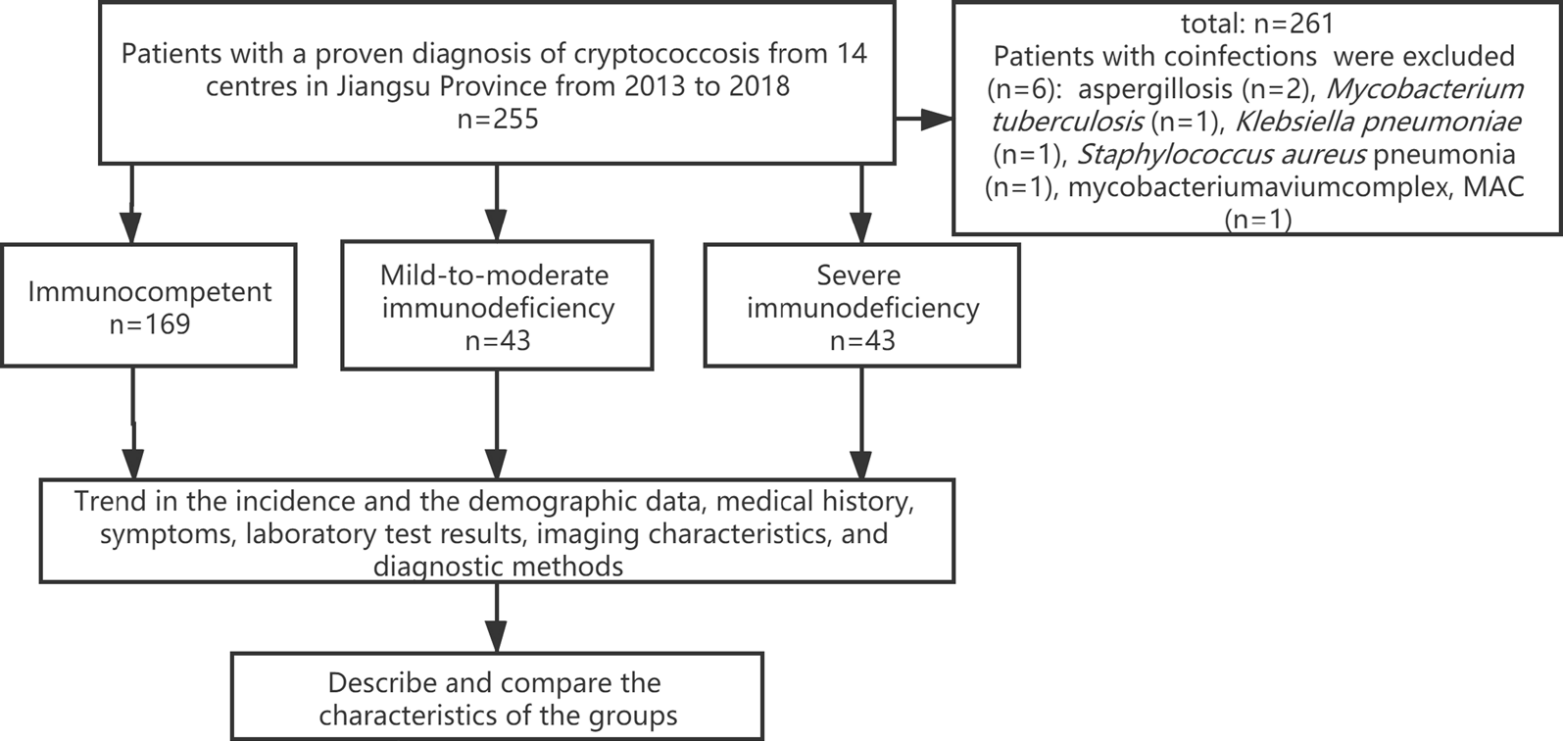
Screening and research flow chart

Proven cryptococcosis cases were reported by 14 tertiary teaching hospitals in Jiangsu Province, China from January 2013 to December 2018. To determine the influence of immune status on the clinical manifestations and diagnostic performance of the CrAg test, patients were carefully evaluated and divided into three groups: the immunocompetent group (IC group), mild-to-moderate immunodeficiency group (MID group) and severe immunodeficiency group (SID group). The patients were also grouped based on whether they had PC or EPC (mainly cryptococcal meningitis). The trend in the incidence and the demographic data, medical history, clinical symptoms, laboratory tests, imaging characteristics and diagnostic methods were analysed and compared (see Fig. 1). The Ethics Committee of Jinling Hospital approved the research protocol. The need of informed consent was waived by the Ethics Committee of Jinling Hospital. None of the data could be traced back to an identifiable patient.

### Criteria for diagnosis and grouping

#### Diagnostic and inclusion criteria

Patients with indicative clinical and imaging manifestations, positive test results for cryptococcal capsular polysaccharide antigen (the name of the kit: CrAg LFA, CRYPTOCOCCAL ANTIGEN LATERAL FLOW ASSAY; the name of the company/manufacturer: IMMY-2701 Corporate Centre Dr Norman Oklahoma 73069 USA—(405) 360–4669; REF: CR2003) in the peripheral blood or cerebrospinal fluid (CSF), or evidence on histopathologic biopsy, or positive results of culture or microscopic examinations [15].

### Exclusion criteria

Patients with co-infections with other pathogens were excluded.

### Grouping criteria:

#### 1) Immune status:

*IC* patients with no recognized underlying disease or those with an underlying disease that does not influence immunity, such as hypertension.

*MID* patients with diabetes mellitus, end-stage liver or kidney disease, autoimmune diseases treated with low-dose glucocorticoid therapy, and cancer treated with chemotherapy.

*SID* patients with AIDS, haematologic malignancies, solid organ transplantation or haematologic stem cell transplantation, idiopathic CD4 lymphocytosis, agranulocytosis, aggressive glucocorticoid or immunosuppressive therapy and other conditions or treatments that result in severe immunosuppression.

The standard of low-dose glucocorticoid is less than 20 mg of prednisone per day, and the cumulative dose is less than 700 mg within 3 weeks before enrollment.

#### 2) Pulmonary and extrapulmonary cryptococcosis:

*PC* Lesions were confined to the lung, no dissemination.

*EPC* Cryptococcal meningitis or cryptococcemia, with or without pulmonary cryptococcosis.

### Statistical analysis

SPSS 26.0 (SPSS Inc., Chicago, IL, USA) were used to perform the statistical analysis and generate the charts in this study. The independent sample T-test was used for continuous variables with normal distributions, and the results are expressed as the means ± standard deviations. The Mann–Whitney test was used for continuous variables with non-normal distributions, and the results are expressed as the medians and IQRs (interquartile ranges). Univariate analysis of variance and the Brown-Forsythe, Welch and Kruskal–Wallis tests were used for multiple groups. The chi-square test and Fisher’s exact test were used for categorical variables. P < 0.05 was considered statistically significant, and logistic regression was used as a multivariate analysis to determine the risk factors.

## Results

### Incidence trend

**Fig. 2:**
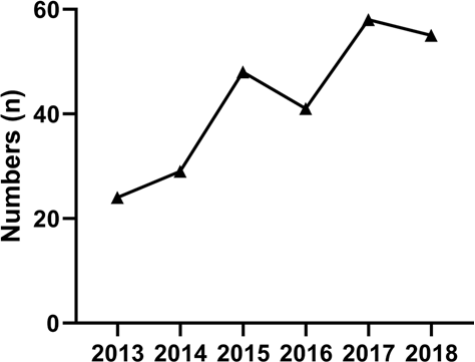
Total number of new cases per year in multiple research centers in Jiangsu Province from 2013 to 2018

Six patients with co-infections were excluded (aspergillosis, n = 2; *Mycobacterium tuberculosis*, n = 1; *Klebsiella pneumoniae*, n = 1; *Staphylococcus aureus* pneumonia, n = 1; mycobacterium complex [MAC], n = 1). Finally, 255 patients with confirmed cases were enrolled in the study. The number of newly proven cases are on the rise overall (24, 29, 48, 41, 58 and 55 from 2013 to 2018, respectively) (see Fig. 2).

### Demographic data

In total, 66.3% of the patients with proven cases (169) were IC, 16.9% (43) were MID, and 16.9% (43) were SID. Of the patients with confirmed cases, 62.7% were males. The mean age was 46.6 ± 13.7 years. Patients in the SID group had a lower body mass index (BMI) [20.2 ± 2.4 kg/m^2^ vs. 23.8 ± 3.0 kg/m^2^ (IC) vs. 24.3 ± 3.4 kg/m^2^ (MID), p < 0.001], although there was no significant difference between the IC and MID groups. Only 6.3% of the patients had a clear history of exposure to bird or pigeon droppings before onset.

Overall, 77.6% (198) of the patients had PC, and 22.4% (57) had EPC. In the IC group, 89.9% of the patients (152) had PC, and 10.1% of the patients (17) had EPC. In the MID group, 81.4% of the patients (35) had PC, and 18.6% of the patients (8) had EPC. However, in SID group, the majority of patients (74.4%, 32 cases) were EPC, and 25.6% of the patients (11) were PC. There was no significant difference in the proportion of patients with MID between the PC and EPC groups (17.2% vs. 15.9%, p = 0.806).

### Medical history

The common underlying diseases in MID group were diabetes (46.5%), receiving low-dose glucocorticoid therapy (25.6%), liver dysfunction (18.6%), chemotherapy (14.0%), autoimmune diseases (14.0%), other renal disease (4.7%) and haemopathy (2.3%) respectively (see Table 1).

**Table 1:** Demographic data and medical history of cryptococcosis patients with different immune statuses

	Total N = 255 (%)	IC N = 169 (%)	MID N = 43 (%)	SID N = 43 (%)	P value
Demographic data					
Sex (male)	160 (62.7)	113 (66.9)	24 (54.5)	23 (54.8)	0.162
Age (y)	46.6 ± 13.7	46.4 ± 12.8	51.8 ± 14.4	41.9 ± 14.9	< 0.001 (#)
BMI, kg/m^2^	23.3 ± 3.3	23.8 ± 3.0	24.3 ± 3.4	20.2 ± 2.4	< 0.001 (*)
Contact history	16 (6.3)	12 (7.1)	3 (7.0)	1 (2.3)	0.504
Time since diagnosis(d)	30.0 (14.3, 60.0)	30.0 (15.0, 60.0)	30.0 (22.0, 60.0)	17.5 (9.0, 30.0)	< 0.001 (*)
Dissemination	57 (22.4)	17 (10.1)	8 (18.6)	32 (74.4)	< 0.001 (*)
Medical history					
Diabetes 13 (6.4) 8 (14.6) 0.06	25 (9.8)	0	20 (46.5)	5 (11.6)	
Glucocorticoid 38 (69.1)	42 (16.5)	0	11 (25.6)	31 (72.1)	
Liver dysfunction	11 (4.3)	0	8 (18.6)	3 (7.0)	
Chemotherapy 05 (9.1)	6 (2.4)	0	6 (14.0)	0	
Autoimmune diseases 0 27 (49.1)	18 (7.1)	0	6 (14.0)	12 (27.9)	
Other renal disease 1 (0.5) 4 (7.3)	8 (3.1)	0	2 (4.7)	6 (14.0)	
Haemopathy	6 (2.4)	0	1 (2.3)	5 (11.2)	
AIDS 08 (14.6)	8 (3.1)	0	0	8 (18.6)	
Kidney transplant 013 (23.6)	13 (5.1)	0	0	13 (30.2)	
Immunosuppressant	31 (12.2)	0	0	31 (72.1)	

Haemopathy included myelodysplastic syndrome (MDS, n = 2), allergic purpura (n = 1), multiple myeloma (MM, n = 1), lymphoma (n = 1), and haemolytic anaemia (HA, n = 1); Autoimmune diseases included systemic lupus erythematosus (SLE, n = 10), rheumatoid arthritis (RA, n = 3), connective tissue disease (CTD, n = 1), sarcoidosis (n = 1), ankylosing spondylitis (AS, n = 1), Hashimoto thyroiditis (HT, n = 1), and pemphigoid (n = 1); Other renal diseases included nephrotic syndrome (NS, n = 6), IgA nephropathy (IgAN, n = 1), and renal cell carcinoma (RCC, n = 1); Liver dysfunction included hepatic cirrhosis (n = 9) and hepatocellular carcinoma (HCC, n = 2)

BMI, body mass index; *SID vs. IC/MID; ^#^MID vs. IC/SID;

### Clinical symptoms

**Fig. 3:**
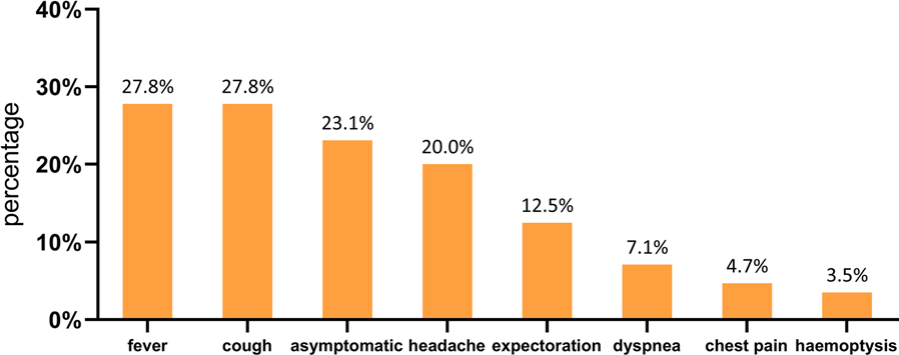
Distribution of clinical symptoms in 255 patients

The clinical symptoms of cryptococcosis are shown in Fig. 3.

Overall, 27.8% (71/255) of the patients had a fever, and the difference among the IC, MID, and SID groups was statistically significant (14.8% vs. 37.2% vs. 69.8%, p < 0.001). Fever was more common in EPC patients than in PC patients (75.4% vs. 14.1%, p < 0.001).

In total, 23.1% (59/255) of the patients presented as asymptomatic, without significant difference in the proportions among the groups [27.2% (IC) vs. 14.0% (MID) vs. 16.3% (SID), p = 0.093]. The proportion of patients with asymptomatic infection was higher in the PC group than in the EPC group (27.3% vs. 8.8%, p = 0.005) (see Table 2). The proportion of patients with headache was 88.2% in the group with cryptococcal meningitis and 3.0% in PC patients.

**Table 2:** Clinical symptoms of cryptococcosis patients with different immune statuses

	Total N = 255 (%)	IC N = 169 (%)	MID N = 43 (%)	SID N = 43 (%)	P value
Fever	71 (27.8)	25 (14.8)	16 (37.2)	30 (69.8)	< 0.001
Cough	71 (27.8)	43 (25.4)	19 (44.2)	9 (20.9)	0.027 (#)
Asymptomatic	59 (23.1)	46 (27.2)	6 (14.0)	7 (16.3)	0.093
Headache	51 (20.0)	17 (10.1)	8 (18.6)	26 (60.5)	< 0.001 (*)
Expectoration	32 (12.5)	22 (13.0)	6 (14.0)	4 (9.3)	0.770
Dyspnea	18 (7.1)	7 (4.1)	5 (11.6)	6 (14.0)	0.036 (&)
Chest pain	12 (4.7)	8 (4.7)	2 (4.7)	2 (4.7)	> 0.999
Haemoptysis	9 (3.5)	6 (3.6)	2 (4.7)	1 (2.3)	0.833
Meningeal irritation	6 (2.4)	0	3 (7.0)	3 (7.0)	0.008 (&)

^*^SID vs. IC/MID; ^#^MID vs. IC/SID; ^&^IC vs. MID/SID

### Laboratory tests

Patients in the SID group had lower levels of haemoglobin and albumin than the patients in the other groups (p < 0.001). Inflammatory markers were slightly elevated in the SID group (C-reactive protein, procalcitonin), although there were no significant differences between the IC and MID groups. The mean white blood cell were in the normal range.

The lymphocyte count in the total population was 1.7 ± 0.9 × 10^9^/L, 2.1 ± 0.8 × 10^9^/L in IC group, 1.5 ± 0.7 × 10^9^/L in MID group, and 0.7 ± 0.4 × 10^9^/L in SID group. The difference between the two groups was statistically significant (P < 0.001) (see Table 3).

**Table 3:** Laboratory test indicators of cryptococcosis patients with different immune statuses

	Total N = 255 (%)	IC N = 169 (%)	MID N = 43 (%)	SID N = 43 (%)	P value
Hb (g/L)	127.9 ± 29.4	137.0 ± 19.5	129.7 ± 36.9	95.7 ± 26.6	< 0.001
WBC (*10^9^/L)	7.1 ± 2.8	7.3 ± 2.8	6.4 ± 2.6	7.1 ± 3.2	0.222
LY (*10^9^/L)	1.7 ± 0.9	2.1 ± 0.8	1.5 ± 0.7	0.7 ± 0.4	< 0.001
Plt (*10^9^/L)	209.3 ± 72.7	221.9 ± 67.3	205.0 ± 72.5	176.3 ± 76.5	< 0.001 (*)
CRP (mg/dl)	2.7 (0.5, 12.9)	1.3 (0.5, 8.2)	2.8 (0.6, 13.8)	14.3 (3.8,41.1)	0.044 (*)
PCT (µg/L)	0.04 (0.03, 0.06)	0.03 (0.02, 0.05)	0.03 (0.02, 0.07)	0.17 (0.05, 2.40)	0.029 (*)
Alb (g/L)	39.6 ± 7.7	42.7 ± 4.9	37.3 ± 7.6	31.5 ± 9.1	< 0.001
APACHE II	4.3 ± 4.7	2.7 ± 3.2	5.0 ± 4.5	9.7 ± 5.5	< 0.001

Hb, haemoglobin; WBC, white blood cells; LY, lymphocyte; Plt, platelets; CRP, C-reactive protein; PCT, procalcitonin; Alb, albumin; APACHE II, acute physiology and chronic health evaluation; CrAg, capsular polysaccharide antigen; *SID vs. IC/MID; ^#^MID vs. IC/SID; ^&^IC vs. MID/SID; () IQR, interquartile range

### Imaging characteristics

**Fig. 4:**
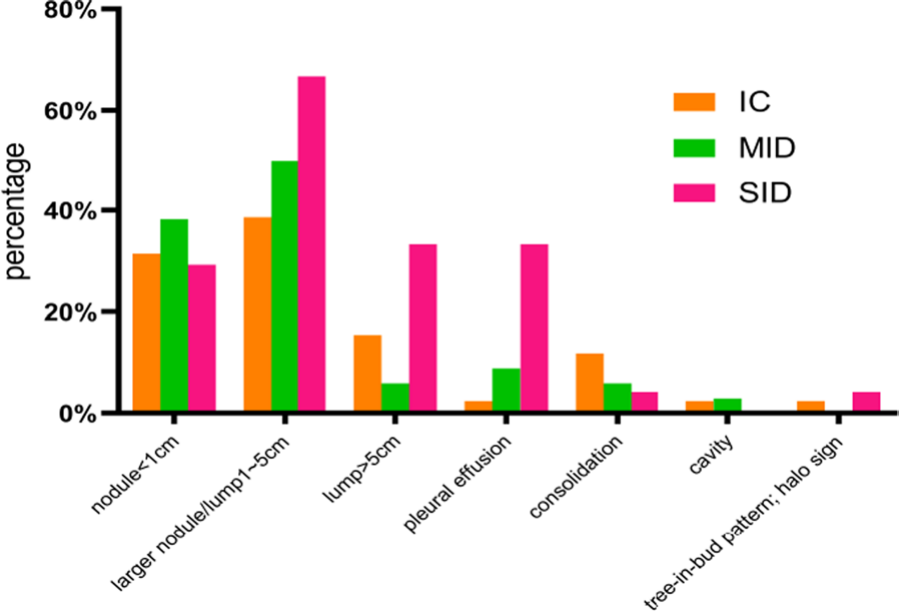
Leisure morphology on imaging of patients with different immune status

The distribution of lesions was classified as solitary (27.5%, 70), multiple (65.5%, 167) or diffuse (7.1%, 18). A subpleural distribution was predominant in all three groups (IC group 72.8% vs. MID group 64.7% vs. SID group 83.3%, p = 0.292), and the remainder were near the hilar or medial belt (see Table 4). The morphology of PC lesions was mainly nodules/lumps (90.3%) (see Fig. 4).

**Table 4:** Lesion distribution and morphology on imaging of patients with different immune status

	Total N = 255 (%)	IC N = 169 (%)	MID N = 43 (%)	SID N = 43 (%)	P value
Distribution (1)					
Solitary	70 (27.5)	55 (32.5)	10 (23.3)	5 (11.6)	0.007 (SID vs. IC)
Multiple	167 (65.5)	107 (63.3)	31 (72.1)	29 (67.4)	0.534
Diffuse	18 (7.1)	7 (4.1)	2 (4.7)	9 (20.9)	< 0.001 (*)
Distribution (2)					
Subpleural	157/216 (72.7)	115/158 (72.8)	22/34 (64.7)	20/24 (83.3)	0.292
Hilar or medial belt	59/216 (27.3)	43/158 (27.2)	12/34 (35.3)	4/24 (16.7)	
Morphology					
Nodule < 1 cm	73/216 (33.8)	53/158 (31.4)	13/34 (38.2)	7/24 (29.2)	
Nodule/Lump 1–5 cm	94/216 (43.5)	61/158 (38.6)	17/34 (50.0)	16/24 (66.7)	0.025 (SID vs. IC)
Lump > 5 cm	28/216 (13.0)	26/158 (15.4)	2/34 (5.9)	0	0.028 (SID vs. IC)
Consolidation	23/216 (10.7)	20/158 (11.8)	2/34 (5.9)	1/24 (4.2)	
Cavity	5/216 (2.3)	4/158 (2.4)	1/34 (2.9)	0	
Tree-in-bud pattern; halo sign	5/216 (2.3)	4/158 (2.4)	0	1/24 (4.2)	
Pleural effusion	15/216 (6.9)	4/158 (2.4)	3/34 (8.8)	8/24 (33.3)	< 0.001 (*)

^*^SID vs. IC/MID

**Fig. 5:**
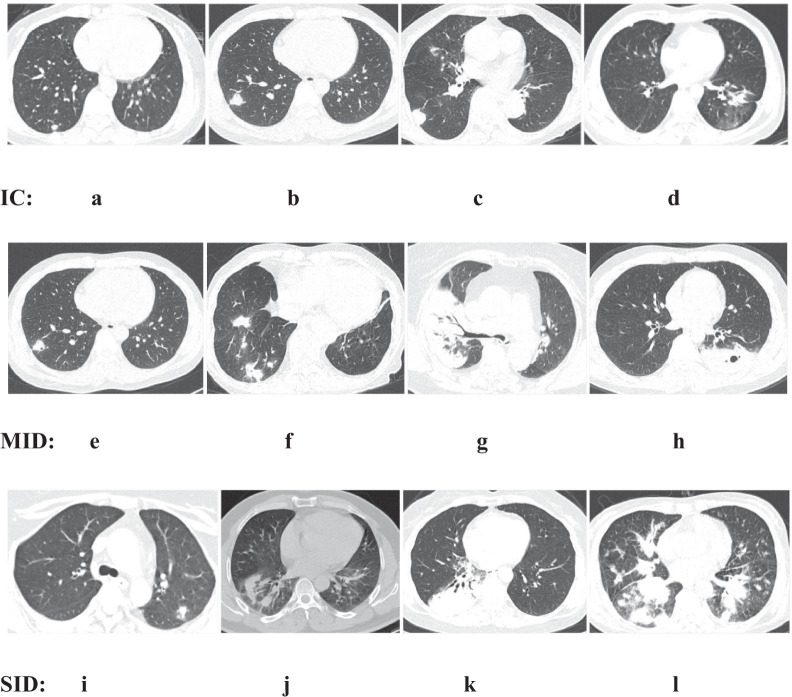
Typical chest computed tomography scans of cryptococcosis patients. IC: **a**-**c**: solitary subpleural nodules of varying sizes in the right lower lung (< 3 cm); **d** irregular nodular shadows in the left lower lung; MID: **e** solitary subpleural nodule with a cavity in the right upper lung; **f** multiple nodular shadows close to the periphery in the right lung; **g** consolidation of the right lung with air bronchogram sign; **h** lump in the lower left lung, with a cavity in the middle; SID: **i** solitary subpleural nodule in the left lung; **j** irregular nodular shadows in the right lower lung; **k** massive consolidation of the right lung with air bronchogram sign and a small amount of pleural effusion; **l** lump in the right lower lung with nodules on the other lobes

We present several representative imaging findings in Fig. 5.

Subpleural nodules were dominant in the IC group, while consolidation shadows and cavities were found in MID group, and more irregular mass shadows and large-scale consolidation shadows were found in SID group.

### Smear microscopic examination

The positivity rate of peripheral blood smear microscopic examination in patients with cryptococcosis was 4.5% (3/66). The positive cases in IC, MID and SID groups accounted for 0/21, 0/15 and 3/30 (10.0%) respectively. In patients with cryptococcal meningitis, the positivity rate of cerebrospinal fluid smear microscopic examination was 68.6% (35/51), and positivity rates in IC, MID and SID groups were 35.3% (6/17), 6/8 and 88.5% (23/26) [P < 0.001 (SID vs. IC)] (see Table 5).

**Table 5:** Smear, capsular polysaccharide antigen and pathology results of patients with different immune status

	Total N = 255 (%)	IC N = 169 (%)	MID N = 43 (%)	SID N = 43 (%)	P value
Smear					
Peripheral blood	3/66 (4.5)	0/21	0/15	3/30 (10.0)	0.544
CSF (meningitis)	35/51 (68.6)	6/17 (35.3)	6/8 (75.0)	23/26 (88.5)	< 0.001(SIDvs.IC)
CrAg					
Serum	128/155 (82.6)	79/101 (78.2)	27/32 (84.4)	22/22 (100.0)	0.048 (*)
Serum (meningitis)	21/21 (100.0)	5/5 (100.0)	2/2 (100.0)	14/14 (100.0)	
CSF (meningitis)	44/45 (97.8)	11/11 (100.0)	7/8 (87.5)	26/26 (100.0)	0.094
Pathology	147/163 (90.2)	119/129 (92.3)	24/29 (82.8)	4/5 (80.0)	0.222
Percutaneous lung puncture	117/130 (90.0)	95/104 (91.4)	18/21 (85.7)	4/5 (80.0)	0.551
Pneumonectomy	31/31	25/25	5/5	1/1	
TBLB	6/13 (46.1)	5/10 (50.0)	1/3 (33.3)	0	

CSF, cerebrospinal fluid; *SID vs. IC/MID;

### CrAg

The positivity rate of the serum CrAg test in the SID group was higher than those in the other two groups [100.0% vs. 84.4% (MID) vs. 78.2% (IC), p = 0.013]. There was no significant difference between IC and MID group. Among patients with cryptococcal meningitis, the positivity rates of the CSF CrAg test were 100.0%, 87.5%, and 100.0% in the IC, MID and SID groups, respectively (p = 0.094), and the positivity rates of the serum CrAg test were 100.0% whatever the immune status. The positivity rate of the serum CrAg test was also higher in cryptococcal meningitis patients than in PC patients (100.0% vs. 79.5%, p = 0.015) (see Table 6).

**Table 6:** Clinical characteristics of pulmonary and extrapulmonary cryptococcosis patients

	Pulmonary N = 198 (%)	Extrapulmonary N = 57 (%)	P value
Site			
Only skin	–	1/57 (1.8)	
Only bloodstream	–	5/57 (8.8)	
Only CNS	–	42/57 (73.7)	
Bloodstream and CNS	–	9/57 (15.8)	
Laboratory test indicators			
LY (*10^9^/L)	2.0 ± 0.8	1.0 ± 0.9	< 0.001
CSF CrAg (meningitis)	–	44/45 (97.8)	
Serum CrAg	105/132 (79.5)	23/23 (100.0)	0.015
Symptom			
Asymptomatic	54 (27.3)	5 (8.8)	0.005
Fever	28 (14.1)	43 (75.4)	< 0.001
Headache/dizziness	6 (3.0)	45 (79.0)	< 0.001
Meningeal irritation	0	6 (10.5)	< 0.001
Medical history			
Mild-to-moderate immunodeficiency	34 (17.2)	9 (15.9)	0.806
Severe immunodeficiency	1246 (6.1)	3140 (54.4)	< 0.001
Glucocorticoid	17 (8.6)	25 (43.9)	< 0.001
Immunosuppressants	10 (5.1)	21 (36.8)	< 0.001
Autoimmune-related diseases	6 (3.0)	12 (21.1)	< 0.001
Kidney transplant	4 (2.0)	9 (15.8)	< 0.001
AIDS	1 (0.5)	7 (10.5)	< 0.001
Diabetes	20 (10.1)	5 (8.7)	0.766
APACHE II, median (IQR)	2.0 (1.0, 5.0)	7.0 (3.5, 10.8)	< 0.001

CNS, central nervous system; LY, lymphocyte

### Pathology

In this study, 174 patients underwent lung histological examination, including 130 cases of percutaneous lung puncture, 31 cases of surgical pneumonectomy, and 13 cases of transbronchial lung biopsy (TBLB), with positivity rates of 90.0% (117/130), 100% (31/31), and 46.1% (6/13), respectively (see Table 5).

## Discussion

The number of newly diagnosed cases of cryptococcosis in Jiangsu Province is on the rising. Most of them are patients with immunocompetency. We analyzed the reasons as follows:

Firstly, the sensitivity of serum CrAg test was high compared with galactomannan and 1-3-β-d-glucan test. The serum sensitivity of patients with normal immune function was 78.2%. A study concluded that the positivity rate for CrAg in the serum was 80.3% in IC hosts [16]. The CrAg test is playing an increasingly important role in clinical practice. According to the 2019 EORTC/MSG, a positivity test for CrAg can serve as confirmatory evidence in the diagnosis of cryptococcosis. This greatly improves the diagnostic status of serum CrAg detection. It is widely used as a routine screening for suspected lung infections. With advancements in detection technology and improvements in clinical diagnostic methods, an increasing number of cases of cryptococcosis with immunocompetency have been discovered [17].

Secondly, minimally invasive techniques such as percutaneous pulmonary puncture are gaining popularity. Immunocompetent patients without CNS/bloodstream/skin involvement were more likely to present as asymptomatic, and lesions were often found on chest CT scan by physical examination [18]. In our study, 27.2% patients presented with asymptomatic infection. In the past, we usually followed up these patients, and the diagnosis was made when patients with immunocompromised immune system developed extrapulmonary spread or presented with respiratory or systemic symptoms. We can now diagnose early with techniques like percutaneous lung puncture or tracheoscopy. At this stage, the majority of patients are with immunocompetency.

In addition, it is related to the increased understanding and diagnostic awareness of clinicians in recent years. It could also have something to do with the increased incidence of the disease itself.

In this study, 77.6% of patients had PC, and only 16.9% of patients had SID. This is inconsistent with the fact that the majority of previously reported cases were CNS *Cryptococcus* infections. A 10-year (2008–2017) retrospective study from western China concluded that most patients with cryptococcosis were immunocompromised (69.1%, 94/136), and the proportions of patients who were immunocompromised were 55.8% (48/86) in the non-disseminated and 92.0% (46/50) in the disseminated groups, respectively [14]. Another report from Chicago also stated that cryptococcal meningitis was identified in 69.1% of HIV-infected patients, 34.4% of non-HIV-infected, nontransplant patients, and 41.9% of solid organ transplant patients [19].

*Cryptococcus* spores are inhaled primarily through the respiratory tract [7, 20–22]. They accumulate in the alveoli, causing lung infections and then spreading to the central nervous system, bones, skin, prostate and other organs. This also explains why in our study, the number of patients with PC was significantly higher than that with EPC.

After the disease, immunodeficient patients cannot produce enough cytokines such as IL-2 and tumor necrosis factor to confine the disease to the lung, and C*ryptococcus* spores may spread with bloodstream and lymph nodes. The most common site of infection is the central nervous system, which may be caused by the lack of C*ryptococcus* capsular antibody in CSF, the lack of complement activation system against the capsule, the dopamine in CSF conducive to the growth of C*ryptococcus*, and may also be related to the increase of the permeability of the tight connection and the destruction of the integrity of the blood–brain barrier [23].

Patients with SID were most likely to be EPC, followed by patients with MID and IC. When extrapulmonary spread occurs, CrAg sensitivity in serum and CSF can reach almost 100%. Patients with high load of C*ryptococcus* pathogens and high antigen concentration in blood are easy to be detected, which is conducive to rapid and definite diagnosis. However, for people with IC and MID, in order to prevent missed diagnosis, negative results cannot completely exclude the diagnosis, and the patient's immune status should be combined.

We conducted a multi-factor analysis to found that patients with severe immune deficiency, headache symptom are independent risk factors for the development of disseminated. But the Odd Ratio (OR) values and confidence intervals (CI) are large. We analyzed the possible reasons as follows: 1. the proportion of patients with PC and EPC was too large, and the sample size of patients with EPC was small; 2. the observational indicators, especially headache symptoms, were significantly correlated with the outcome and the effect size was large, so the OR value and CI were large. When patients with PC are combined with severe immunodeficiency, headache, central nervous system infection should be considered, and timely lumbar puncture should be performed.

Cryptococcosis mainly occurs in middle-aged men aged 40–50 years, and the male predominance may be related to differences in the immune system and physiology between men and women [6, 24].

Based on the above results, more severely immunodeficient patients are more likely to develop a fever. Similarly, EPC patients were significantly more likely to have a fever than PC patients (75.4% vs 14.1%, p < 0.001), and this result was consistent with a study from Western China (50.0% vs 23.2%, p = 0.001) [14, 25, 26]. We believe that when the immunodeficiency is aggravated, the body does not produce a strong immune response and enough cytokine, like interleukin-2 against *Cryptococcus* infection. Then the pathogen load is high in the body and produce more endogenous pyrogen, through blood circulation effects on the temperature regulating center and change its function, cause fever reaction.

We observed that inflammatory markers, such as CRP protein and PCT, were normal in IC and MID patients and slightly elevated in the SID group. White blood cell were essentially normal in all groups. This suggests that normal inflammatory indicators do not exclude pulmonary cryptococcosis. On the other hand, in the diagnosis and differential diagnosis of disease, if the patient's inflammatory indicators are significantly increased, the presence of other pathogen infection should be further considered.

Multiple subpleural nodules/lumps or consolidations are important imaging features of PC. Patients with SID were more likely to have pleural effusion in this study. These imaging findings have a degree of specificity compared to other pulmonary infections. This also explains why percutaneous lung biopsy is used in most pathological examinations, and the positive rate of percutaneous lung biopsy (90.0%) is much higher than that of TBLB (46.1%). These are closely related to the characteristics of cryptocosis in which the lesions are mostly located in subpleural [12, 16, 27, 28].

The positivity rate of traditional smear microscopic examination was lower than that for the serum CrAg test. However, in cryptococcal meningitis patients with SID, the positivity rate reached 88.5% in CSF, which is nearly similar to the 85% reported in meningitis patients [29]. Therefore, patients with suspected cryptococcal meningitis should be examined by lumbar puncture as soon as possible to obtain a CSF specimen for testing [30, 31].

This study, which uses underlying disease to divide patients into different immune groups, does not seem objective. Part of cases in our study were retrospective, lacking some objective indicators, and there is no single objective indicator to distinguish the systemic immune status of patients. Furthermore, we analyzed the peripheral blood lymphocyte counts of patients and found that the difference in peripheral blood lymphocyte counts between different groups was statistically significant. This also confirms that the classification method we use for underlying diseases is relatively reliable and close to the clinic to facilitate rapid judgment by clinicians. In future studies, if objective immune indicators were used to group patients, such as CD4+ T lymphocyte cell count, it might bring us more valuable information.

## Conclusion

The clinical presentation of MID patients is intermediate between those of SID and IC patients and is similar to those of IC patients in many respects. The serum CrAg test is relatively more sensitive in SID or EPC patients.

## Data Availability

The datasets during and/or analysed during the current study are available from the corresponding author on reasonable request.

## Abbreviations

PC: Pulmonary cryptococcosis
EPC: Extrapulmonary cryptococcosis
IC: Immunocompetent
MID: Mild-to-moderate immunodeficiency
SID: Severe immunodeficiency
CrAg: Capsular polysaccharide antigen
AIDS: Acquired immunodeficiency syndrome
IL-2: Interleukin-2
TNF: Tumor necrosis factor
BMI: Body mass index
IDSA: Infectious Diseases Society of America
EORTC/MSG: European Organization for Research and Treatment of Cancer and the Mycoses Study Group Education and Research Consortium
Hb: Haemoglobin
WBC: White blood cells
NE: Neutrophil
Plt: Platelets
CRP: C-reactive protein
PCT: Procalcitonin
Alb: Albumin
APACHE II: Acute physiology and chronic health evaluation
BALF: Bronchoalveolar lavage fluid
CSF: Cerebrospinal fluid
TBLB: Transbronchial lung biopsy
CNS: Central nervous system
